# Study of dog population dynamics and rabies awareness in Thailand using a school-based participatory research approach

**DOI:** 10.1038/s41598-024-71207-7

**Published:** 2024-09-03

**Authors:** Weerakorn Thichumpa, Anuwat Wiratsudakul, Sarin Suwanpakdee, Chayanin Sararat, Charin Modchang, Setha Pan-ngum, Nakornthip Prompoon, Onpawee Sagarasaeranee, Sith Premashthira, Weerapong Thanapongtharm, Arun Chumkaeo, Wirichada Pan-ngum

**Affiliations:** 1 Department of Tropical Hygiene, Faculty of Tropical Medicine, Mahidol University, Bangkok, Thailand; 2 Department of Clinical Sciences and Public Health, and the Monitoring and Surveillance, Center for Zoonotic Diseases in Wildlife and Exotic Animals, Faculty of Veterinary Science, Mahidol University, Nakhon Pathom, Thailand; 3 Biophysics Group, Department of Physics, Faculty of Science, Mahidol University, Bangkok, Thailand; 4 Centre of Excellence in Mathematics, MHESI, Bangkok, Thailand; 5 Department of Computer Engineering, Faculty of Engineering, Chulalongkorn University, Bangkok, Thailand; 6 Department of Livestock Development, Ministry of Agriculture and Cooperatives, Bangkok, Thailand; 7 Songkhla Provincial Livestock Office, Muang, Songkhla, Thailand; 8 Mahidol Oxford Tropical Medicine Research Unit (MORU), Faculty of Tropical Medicine, Mahidol University, Bangkok, Thailand

**Keywords:** Dog population, Dog owner, Rabies awareness, School-based participatory research (SBPR), Public health, Epidemiology, Diseases, Health care, Risk factors

## Abstract

Rabies is a neglected disease primarily related to dog-mediated transmission to humans. Accurate dog demographic and dynamic data are essential for effectively planning and evaluating population management strategies when designing interventions to prevent rabies. However, in Thailand, longitudinal survey data regarding dog population size are scarce. A school-based participatory research (SBPR) approach was conducted to survey owned dogs for one year in four high-risk provinces (Chiang Rai, Surin, Chonburi, and Songkhla) of Thailand, aiming to understand dog population dynamics and raise awareness about rabies. ‘Pupify’ mobile application was developed to collect data on dog population and observe the long-term population dynamics in this study. At the end of the data collection period, telephone interviews were conducted to gain insight into contextual perceptions and awareness regarding both animal and human rabies, as well as the social responsibility of dog owners in disease prevention and control. Among 303 high school students who registered in our study, 218 students reported at least one update of their dog information throughout the one-year period. Of 322 owned dogs from our survey, the updates of dog status over one year showed approximately 7.5 newborns per 100-dog-year, while deaths and missing dogs were 6.2 and 2.7 per 100-dog-year, respectively. The male to female ratio was approximately 1.8:1. Twenty-three students (10%) voluntarily participated and were interviewed in the qualitative study. The levels of rabies awareness and precautions among high-school students were relatively low. The high dropout rate of the survey was due to discontinuity in communication between the researcher and the students over the year. In conclusion, this study focused on using the SBPR approach via mobile application to collect data informing dog population dynamics and raising awareness regarding rabies in Thailand Other engaging platforms (e.g. Facebook, Instagram, Twitter, and other popular applications) is necessary to enhance communication and engagement, thereby sustaining and maintaining data collection. Further health education on rabies vaccination and animal-care practices via social media platforms would be highly beneficial. For sustainable disease control, engaging communities to raise awareness of rabies and increase dog owners’ understanding of their responsibilities should be encouraged.

## Introduction

Population demographics are important baseline data necessary for the study of infectious diseases. Human population data are available in most settings. For animal populations, however, demographic information is very limited in several countries and often only available for specific cohorts or studies. In Thailand, nationwide dog surveys are conducted by local government organizations once or twice a year and reported to a web-based reporting system, “ThaiRabies.net”, which has been updated to “Rabies One Data” since 2021^1^. These surveys require considerable human resources, while the quality of data can vary from province to province depending on the management and training of local staff teams to process and manage data^2^. Here, we proposed an innovative way to conduct dog surveys using a school-based participatory research (SBPR) as a part of community-based participatory research (CBPR), an approach to research that involves collective, reflective, and systematic inquiry in which researchers and community stakeholders engage as equal partners in all steps of the research process, with the goal of educating, improving practice, or bringing about social change^3,4^. We implemented the SBPR approach to perform a dog population survey among high school students in Thailand, using a mobile-phone application. This alternative approach relies on a self-reporting system for dog owners. This can be done through a mobile application developed for data collection. This approach was hoped to provide solution of a long-term data collection with lower cost to the government sectors, as well as promote community participations, raising awareness and responsibility among owners to register, monitor, and care for their dogs.

Dog ownership issues are critical for the design of rabies vaccination campaigns, especially in developing countries, including Thailand^5^. In many high-income settings, owners are responsible for properly confining their dogs and facilitating their vaccination against rabies. In Thailand, dog-keeping practices and duties of responsible ownership vary depending on the cultural setting^6^. There is an increasing evidence that most free-roaming dogs are owned and accessible for rabies prophylaxis^7–9^; moreover, unvaccinated owned dogs have been affected by rabies^2^. Nevertheless, many owners cannot afford to pay for vaccination and other veterinary care for their own dogs^10,11^. Thus, many people rely on free, mass vaccination campaigns against rabies, provided by the government or non-governmental organizations (NGOs). In addition, limited access to dog vaccination can potentially reduce effective vaccination coverage, particularly if the proportion of unowned dogs is large. Dog movement patterns can also play a role in rabies epidemiology^12^. Dog confinement has been studied and implemented in some countries as a control measure for rabies^13–15^.

In Thailand, rabies is a notifiable condition, however it is not compulsory to report suspected rabies exposure in humans^16^. Both dog and human vaccination guidelines from the World Health Organization (WHO) and the World Organization for Animal Health (WOAH), recommend a comprehensive strategy to eradicate dog-mediated rabies^17–19^. The strategy highlights the importance of mass dog vaccination campaigns (aiming for at least 70% coverage) and the implementation of effective dog population control measures (e.g. sterilization), which have been optimized for rabies prevention and control^16–18,20^. Human rabies in Thailand has been prevented and controlled by policy promulgated since 1992. Rabies cases have decreased because of schemes including mass dog vaccination and sterilization. Although human rabies in Thailand has gradually declined, animal rabies has been generally increasing over the past ten years^2^. In 2020, there were 209 cases of rabid dogs reported in Thailand and three human deaths due to rabies. Rabies is most prevalent in the provinces of Chonburi, Songkhla, and Surin, while Chiang Rai has found high positive detection of rabid animal cases in 2018^21,22^. The control of rabies in animals is challenging, as the disease can be transmitted throughout the year and therefore surveillance and control of animal carriers are urgently required^20^. As for the Thai government’s policy and guideline (based on WHO & WOAH) for high-risk areas, ring vaccination is currently implemented for controlling and preventing rabies outbreaks, while sterilization is a long-term solution to control number of dog population, reducing contacts among dogs and between human and dogs. Both vaccination and sterilization are hopeful for improve management of dog bites^22^.

Although the database of dogs has been significantly improved following the introduction of dog survey reporting to ThaiRabies.net by local government organizations, the system still relies solely on the public health sectors. Moreover, data consistency remains an issue due to technical problems within the system and incomplete data entry. Here, we introduced a novel method for owned-dog data collection, using the SBPR approach. Information about dog population dynamics is essential for analyzing population and disease prediction and can act as baseline data for dog population management plans. The exploration and identification of dog population ecosystems and dynamics are required as a framework to effectively plan and evaluate population management and interventions to prevent rabies^8^. In addition, the introduction of an approach to our dog survey among school-age children could be beneficial in terms of generating awareness of animal-care practices, disease, and the development of a research mindset.

Countries in Southeast Asia are among the top users of mobile phones globally. In 2020, total population of Thailand were approximately 65.42 million^23^. The number of smartphone users in Thailand reached 53.57 million, with around 60 million predicted by 2026, due both to increases in the Thai population and internet penetration^24^. Self-reported data collection via mobile phones can be of use when conducting large-scale surveys, with the affordability and availability of mobile phones and wireless networks making them a viable alternative to traditional methods^25^. However, it is important to consider various aspects involved in the development and implementation of mobile phone data collection. For example, ensuring usability and user acceptance of the data collection system will help motivate survey participants to stay with the project and continue to provide high-quality data. Server authentication through the use of properly configured certificates will help deal with threats of data submission to a malicious server, which can increase users’ confidence in data security^26^.

Our study proposed an initial effort to conduct a long-term survey based on dog owners’ awareness and participation. The dog population dynamics data were analyzed and visualized. In addition, the qualitative study was performed on 10% of the survey participants who volunteered to do the interview on knowledge of rabies, social responsibility, community engagement and research orientation. The data collection tools and methods were assessed and further improvements when using this approach were proposed.

## Results

### Dog population survey

#### School and participant demographics

**Fig. 1:**
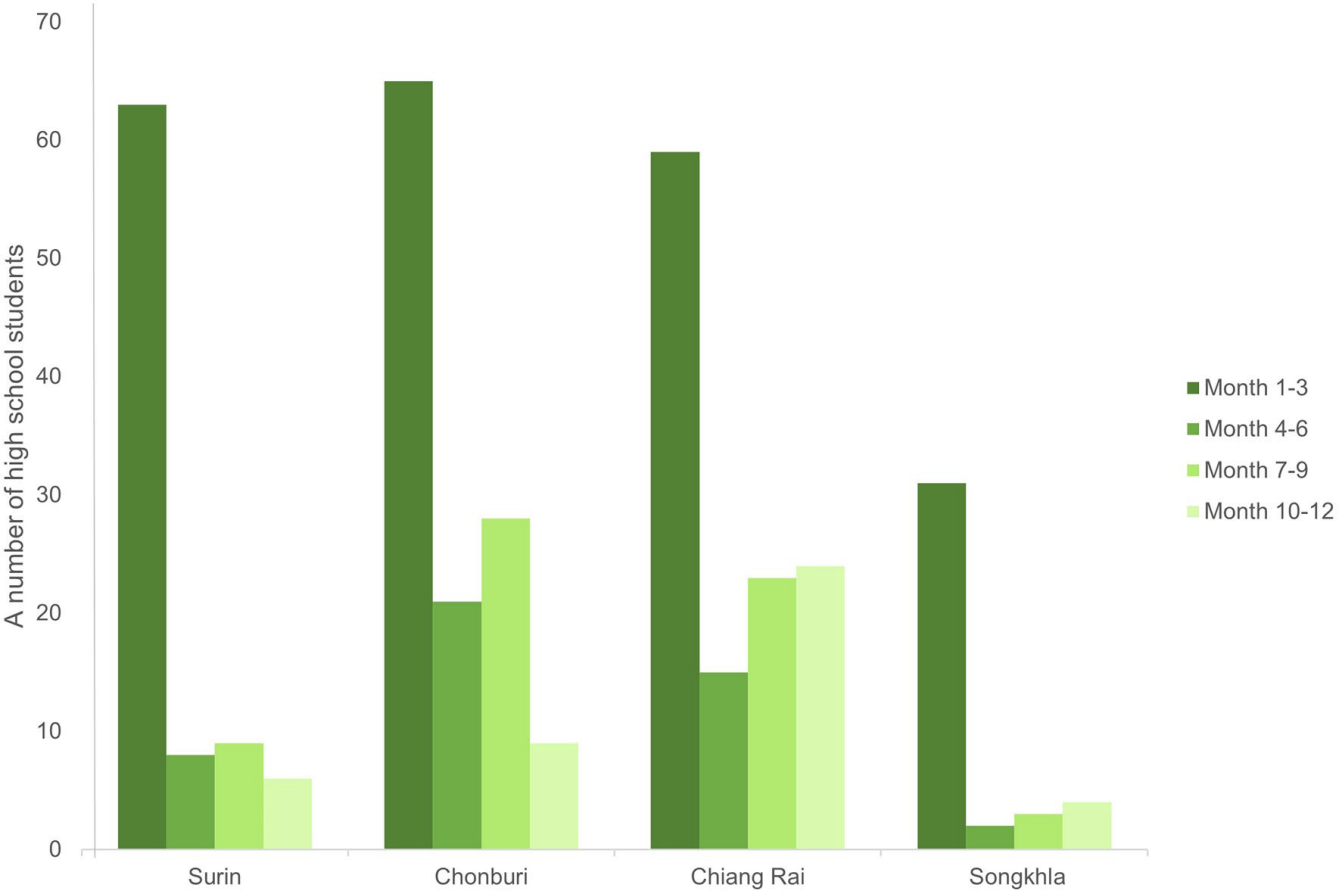
A number of participants’ responses in a 3-month period during the study year.

In the survey via ‘Pupify’ mobile application, 303 high-school students registered through the mobile application for our study. There were 29.8%, 28.9%, 27.1% and 14.2% from a school in Chonburi, Surin, Chiang Rai, and Songkhla provinces, respectively; most were female participants (72.9%) (Table 1). Of 303 registrations, 218 participants actually submitted at least one update of their dogs into the system over the one-year study. However, the number of participants continued submitting the monthly dog updates dropped to 46, 63 and 43 after 6 months, 9 months and by the end of one year, respectively. The number of students giving the completely one-year updates was 43 or 20% of total participants from the start (Fig. 1).

**Table 1:** Characteristics of the participants.

	Number	Percentage
(a) Participants in the survey		
Recruitment*	303	100.0
During the survey	218	72.0
End of the survey	43	19.7
Gender		
Male	59	27.1
Female	159	72.9
School area		
Chonburi	65	29.8
Surin	63	28.9
Chiang Rai	59	27.1
Songkhla	31	14.2
Age range 16–17 years	303	100.0
(b) Participants in the qualitative study (n = 23)		
Gender		
Male	3	13
Female	20	87
Response		
Provided no update (only registration)	4	17.4
Partially updated	9	39.1
Fully updated	10	43.5
School area		
Chonburi	12	52.2
Chiang Rai	7	30.4
Surin	2	8.7
Songkhla	2	8.7

*Participants who registered via ’Pupify’ mobile application.

#### Dog demographics and dog population dynamics

Overall, 322 owned dogs were reported during the study period. More than half were male dogs (65.0%). Owned dogs were divided into three age groups based on owners′ identification: birth to 1 year (28.3%), aged between > 1 and 8 years (57.1%), and aged > 8 years (14.6%). These age classes were used to represent three groups of dogs, puppy, adult, and elderly. Most owned dogs were reported in Surin province (35.4%), followed by Chiang Rai (28.5%), Chonburi (22.7%), Songkhla (12.1%), and others where owners dwelling in adjacent areas (1.2%). In addition, 24 new-born puppies were reported, while there were 20 deaths (e.g. caused by dog illness, bite, fight, accident, and culling) and 9 missing dogs reported. These numbers correspond to the estimated birth, death, and missing rates of 7.5, 6.2, and 2.7 per 100 dog-years, respectively. Based on the self-reporting system, 40.1% of the dogs had been vaccinated against rabies and 12.4% had been sterilized (Table 2).

**Table 2:** Demographic details of owned dogs.

	Number	Percentage
(a) Dog data on registration (n = 301)		
Sex		
Male	196	65.1
Female	105	34.9
Age		
Birth to 1 year	87	28.9
> 1 to 8 years	174	57.8
> 8 years	24	8.0
N/A	16	5.3
Mean = 3.9 years		
Area		
Surin	104	34.5
Chiang Rai	82	27.2
Chon Buri	71	23.6
Songkhla	39	13.0
Others (nearby)	5	1.7
(b) Dog data from monthly updates (n = 322)		
Types		
Confined dogs (in a limited area)	226	70.2
Free roaming dogs	96	29.8
Dynamics		
Newborn	24	7.5
Died	20	6.2
Missing	9	2.7
Interventions		
Rabies vaccination	129	40.0
Sterilization	40	12.4

### Qualitative study

#### Dog owner characteristics

A total of 23 high-school students, all aged 17 years, voluntarily participated in our interview (see Supplementary Table 3). There were students from all three levels of participation, including registration only (17.4%, n = 4), partially updated data (39.1%, n = 9), and fully updated data (43.5%, n = 10). Although all schools from four provinces were represented, more than half of the participants were from Chonburi province (52.2%).

#### Extensive knowledge and dog rabies awareness

Most participants (91.3%, n = 21) strongly agreed that rabies was fatal, resulting in death in both humans and dogs. One participant noted, *“I learned from the news on TV that human infections result in a hundred percent mortality”*. However, 52.1% of the participants (n = 12) reported that they were either unaware of or did not follow rabies situations locally. This indicated that while most participants are aware of rabies, they do not necessarily stay informed about local rabies situations. One participant said, “*I have very little experience of rabies disease. I have not seen the real case before and have not followed the disease situation. At school, there is minimal information for us to research more about rabies. Sometimes, external health staff came to educate us about health at school but didn’t focus on rabies”*. While a majority (65.2%, n = 15) of participants considered that only cats and dogs were reservoirs for rabies, a larger proportion (78.2%, n = 18) were unsure whether there were other animal reservoirs. This result indicated that most participants were unaware that other mammals can also get infected with rabies. From the interviews, some participants made statements such as *“I think it mainly comes from dogs and cats, unlikely to be other species”* and *“Most cases are infected from stray dogs, perhaps also from rabbits and monkeys”*. In addition, 65.0% (n = 15) of participants mainly received information about rabies from social media and other online sources, while the remaining participants obtained information from other sources, including schools (such as our project visit), television and news, community announcements, medical providers, parents, and relatives.

#### Rabies precautions and caring for owned dogs

Most participants (87.0%, n = 20) stated that avoiding contact with stray dogs can help to prevent rabies infection. Also, 52.1% (n = 12) suggested that owned dogs should be vaccinated annually against rabies. Dog confinement was reported by most owners (87.0%, n = 20) as a way to control and limit their dogs’ contact with humans or animals. One participant said, *“I keep my dog only in my house to avoid contacting with people and other dogs”* and *“My dog is always leashed all the time and I don’t allow other dogs nearby my dog when it is outside”*. According to this, half of them (52.1%, n = 12) trusted their dogs, with 80–100% confidence due to annually vaccination and not allowing dogs outside. One participant said, *“Some of my dogs are not yet vaccinated, we put the dogs to guard our properties in the factory area and sometimes outside dogs do come to visit”*.

In terms of caring for owned dogs, participants reported how they managed their dog’s health (including regular health check-ups and visits to veterinarians when health issues were identified). The majority used the services of animal clinics (87.0%, n = 20), followed by animal hospitals (21.7%, n = 5), treatment by owners (21.7%, n = 5), and government veterinary services (13.0%, n = 3). However, one said, *“I saw my aunt giving paracetamol to the dog when it was sick. I didn't agree with that and would have looked for more information or taken the dog to the vet instead”*. This indicated that animal health education on the care of owned dogs should be enhanced, with information provided by specialists at animal service stations.

In the case of what happens to newborn puppies, participants identified two common situations: giving them away to others (65.2%, n = 15) and keeping the puppies themselves (39.1%, n = 9). In the mating season, most participants said they confined their dogs and did not allow them to breed with other dogs. One participant said, *“I usually keep the dog in the house and sometimes use a lease to prevent dogs fighting”.* Conversely, in the case of both neutered and non-neutered dogs, some participants still allowed their dogs to breed. Finally, the owners said they commonly observed their dog’s health status at feeding time (47.8%, n = 11); when they were sleeping (30.4%, n = 7) or playing (17.4%, n = 4); or when they observed any abnormality (17.4%, n = 4).

#### Obstacles, limitations, and motivations for joining in with school-based participatory research

Obstacles and limitations relating to the SBPR study mentioned by participants included forgetting to update their dog’s data (65.2%, n = 15), having school assignments and portfolios (30.4%, n = 7), having a part-time job (17.4%, n = 4), having personal works (17.4%, n = 4), having a poor internet connection (13.0%, n = 3), changing their smartphone (8.7%, n = 2), being unable to install the mobile application (4.3%, n = 1), and not interested in participating (4.3%, n = 1).

Conversely, participants reported some interesting advantages and motivations for why they participated in this study. Motivations included in the attainment of project certificates (60.9%, n = 14), followed by project rewards/gifts (34.8%, n = 8), research experience (13.0%, n = 3), dog care and follow-up (13.0%, n = 3), and rabies information (4.3%, n = 1). Other influences for joining the project mentioned included own self (65.2%, n = 15), project notification (13.0%, n = 3), project rewards (8.7%, n = 2), and support for school activities (4.3%, n = 1). After participated in this study, the main advantages given were mostly focused on caring for owned dogs, with regard to dog attention and care (69.6%, n = 16), observation of dog behavior (34.8%, n = 8), dog vaccine notification (17.4%, n = 4), and education (17.4%, n = 4). One mentioned that *“In my opinion, the best thing I learned is to pay more attention to my dog. I observe my dogs more regularly and take care of them much better than earlier”*.

#### Other suggestions from participants

Some participants suggested that they needed more information about rabies disease, its prevention and control, dog management, and dog vaccination. This could be added to the Pupify application, which was easily accessible for necessary information. Also, alternative sources of information should be considered, e.g., infographics and dog fan-pages on Facebook, Instagram, Twitter, or other popular social media platforms. One participant suggested, *“I think having different channels for communication would help stimulate more interest in the work, for example, forming a ‘dog lovers’ group on social media”*.

## Discussion

Here, we explored a new method to collect dog data via mobile application, a self-reporting system for dog owners, by focus initially on high school students who owned smartphones, which is in contrast with the conventional dog population census that is performed once or twice per year in Thailand by the government departments responsible for animal health. The key challenge to our design was the number of losses to follow-up. Our qualitative study revealed the main barriers to update dog dynamics data were due to some personal issues and technical reasons. A participant from the partial update group noted, *“I gave regular updates until I changed my smart phone, I stopped updating the information completely”*. One from the no-update group said, *“I had difficulties installing the app and I think I am not disciplined enough to join this project anyway”.* In addition, there was some feedback on the suitability of a mobile instant messaging app for data tracing. One participant suggested, *“I prefer other channels of communication such as Instagram and Facebook because they are more convenient to me”*.

Nevertheless, we estimated birth, death, and missing rates of 7.5, 6.2, and 2.7 per 100 dog-years, respectively. The male to female ratio was approximately 1.8:1. The variations in these rates and ratios among the studied provinces are noticeable (see Supplementary Tables 1 and 2). This could be due to different nature of owned dogs in different parts of Thailand. However, due to the relatively small sample size in our study, it would not be appropriate to perform any sub-analysis from this data. It is important to note that the majority of the data provided pertained to confined dogs (70.2%), which may not accurately reflect the uncertainty conditions of free-roaming dogs. Future dog censuses should include a focus on confined, free-roaming, and stray dogs to provide a more comprehensive representation of the overall dog population size. Observations in South Africa revealed that birth and death rates were 31.3–45.1 and 40.6–56.8 per 100 dog-years, respectively, while the male to female ratio was approximately 1.4–1.7:1^27^. A study in India estimated an annual per capita birth and death rate of 1.0 and 0.7, respectively, while the male to female ratio was approximately 1.4:1^28^. A sight–resight survey in Australia reported birth and death rates were approximately 2.4 and 1.7 dogs/dog-owning house/year, respectively, while the male to female ratio was approximately 1:1^7^. Compared with other studies (using different approaches to collect the data; including observational, sight-resight, and/or mark-recapture survey), births and deaths in our study were relatively low. However, the male to female ratio was in line with previous studies. Similarly to a previous study^6^, we found the proportions of dog-keeping approaches (i.e. confined or free-roaming) varied among the sites, with dogs usually confined in well-developed areas whereas free-roaming dogs were reported more frequently in remote areas.

Our study had some limitations. First, the survey was restricted to owned dogs. It would be helpful to collect similar data for stray dogs; however, to conduct a similar study of stray dogs in the Thai setting, individuals who take care of stray dogs, so called “local feeders”, must be identified^29^. Second, the participants only comprised high-school children of a specific age group, perhaps a broader target public population should be considered for future surveys. Furthermore, we simply used three reproductive age classes to represent puppy, adult, and elderly i.e. the exact dog ages as detailed classifications, i.e. puppy, juvenile, young adult, mature adult, senior, and geriatric, are not available in this study. Third, the 'Pupify' application was developed for Android phones only and required updates to remain compatible with the latest operating system versions. Fourth, there was a low number of one-year data completion among the participated students who owned a mobile phone. Because the participation was voluntary, unrelated to school nor teacher’s request. The study sites were distant from the central project location, notifications and encouragement communications were conducted solely via Line messaging application and telephone calls. This led to discontinuities in communication between the researchers and the students throughout the year. The barriers in our SBPR engagement were limitations of the mobile application platform, technical issues, personal reasons, and the lack of engagement of project through the teachers and/or schools. Further studies should consider site visits to enhance communication, encourage participation, and investigate any arising issues.

In accordance with “One Health” concepts, human health is closely connected to the health of animals and our shared environment, and research in this area should be a collaborative, multisectoral, and trans-disciplinary approach to achieve optimal health outcomes. We made considerable effort to use the SBPR approach in conducting this study. In addition, the initial motivation for study participation was primarily driven by the desire to achieve long-term goals and enhance their profiles for university enrollment. After participating, they also recognized considerable benefits in caring for their dogs and demonstrated a commitment to sustainable effort for better dog care. Although there was a low response rate among participants, we could remark that the main advantage concerning caring for owned dogs was initially successful based on participants’ perception. Most interviewees agreed that this study would encourage them to pay more regular attention to their dogs regarding their health, vaccinations, and rabies prevention. Our study demonstrated the importance of encouraging, among school-age children, early learning about the importance of disease prevention and awareness, together with community engagement and social responsibility for their future. Finally, it is important to note that the success of several research depends on effective data collections. However, this study has provided valuable lessons, demonstrating that engaging the general public, beyond researchers and experts, presents considerable challenges. Practical issues such as invitations, communications, cooperations, maintaining engagements, and overall participations should be carefully considered. We hope that the insights gained from our study with SBPR may be beneficial for further studies and similar contexts.

## Conclusions

Using the SBPR approach for collecting dog population dynamics data among the high school students can be challenging. Additionally, this study was conducted with an initial effort to explore the potential of using SBPR for data collection. The primary objective aimed to propose extending the approach beyond student awareness to include general dog owners in further research. Implementing a suitable SBPR approach involves designing educational activities, training participants, conducting surveys, and engaging the community. This could lead to effective and sustained data collection while fostering community involvement and awareness in the future. Perception on the usefulness of the application and different social-media channels for communication should be considered for future development of data collecting tools and mobile application in order to provide higher incentive to participate and update dog information in a long-term. A low level of disease awareness among high school students was identified in the interviews, possibly due to insufficient information, both at school and in the media. It is critical to promote disease awareness through health education. Further studies using in-depth interviews should focus on enhancing rabies awareness, increasing owner responsibility, and supporting rabies prevention projects, as these factors are crucial for policymaking and effective public participation. Nevertheless, by conducting data collection using a new alternative approach among the students, it has clearly increased some awareness on the importance of animal welfare and provided some new experience of being part of a research for some students to reduce rabies among humans and animals.

## Methods

### Study sites and participants

**Fig. 2:**
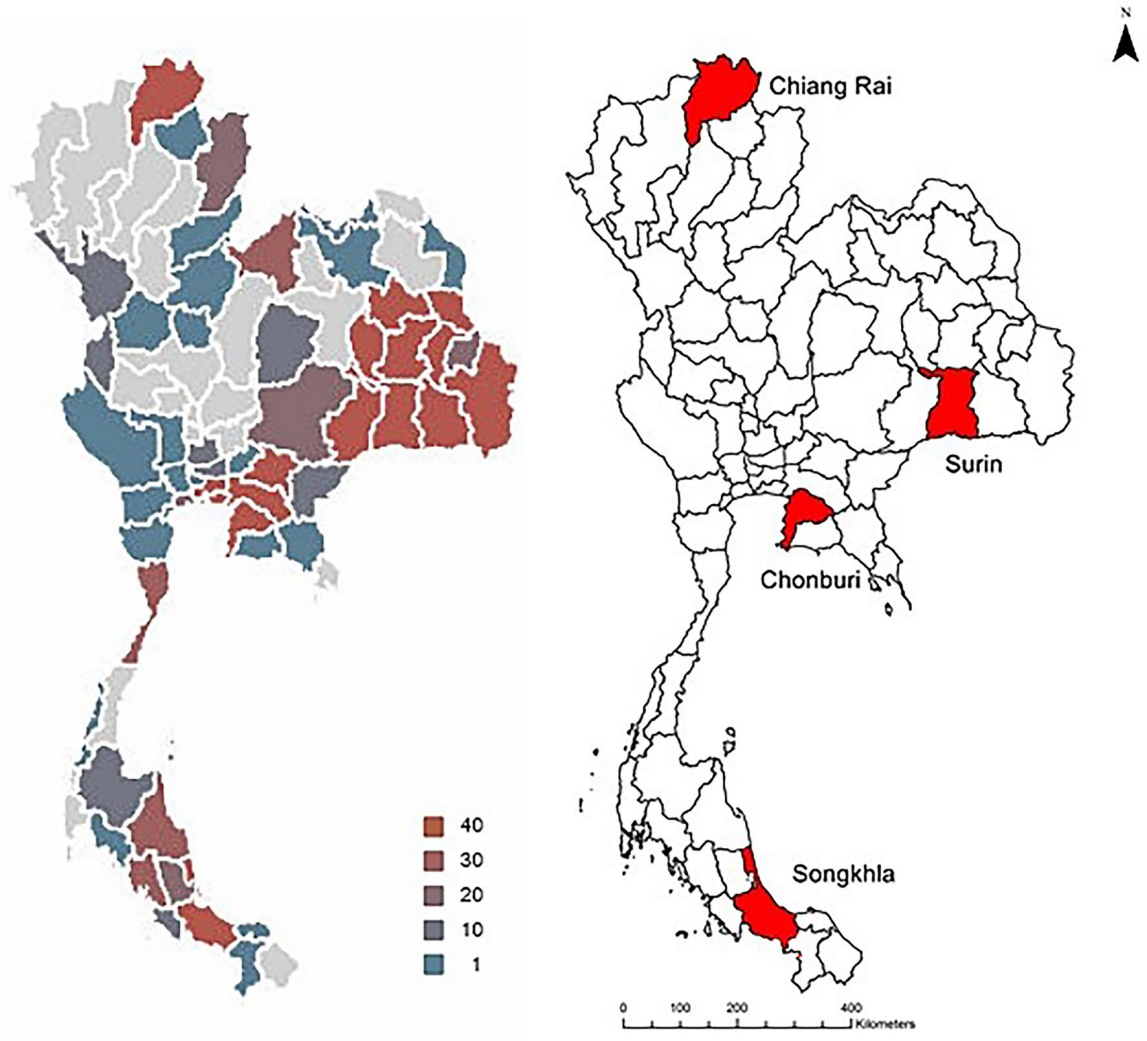
Maps showing; laboratory positive detection of rabies cases in animals in 2018 (Source: Thairabies.net: http://www.thairabies.net^1^; and The four provinces included in the study: Chiang Rai, Surin, Chonburi, and Songkhla.

This study was conducted between June 2018 and October 2019, in areas where rabies is endemic and where there is a high incidence of animal and human cases^30^. It formed part of a larger study conducted in Thailand between 2015 and 2018, which aimed to investigate the cultural and socioeconomic factors that contribute to rabies outbreaks in Thailand^31^. Four provinces were included: Chiang Rai province in the north, Surin province in the northeast, Chonburi province in the east, and Songkhla province in the south (Fig. 2). Based on the past five year report of rabies in Thailand^22,30^, we purposively surveyed high school students dwelling in high endemic areas among the four provinces. Inclusion criteria were: (1) students aged between 16 and 17 years who owned at least one dog and possessed a smartphone that used the Android operating system, and (2) volunteer students whose parents consented to their participation in the study. In this study, dog ownership was defined as those who owned or cared for at least one dog at the residence only. Students were eligible to voluntarily participate by registering dog data on the ‘Pupify’ application.

#### Data collection using the “Pupify”

‘Pupify’ mobile application was developed to collect long-term data on dog population numbers and dynamics from dog owners, feeders, and the general public. The ‘Pupify’ was developed by a group of university students from the Department of Computer Engineering, Chulalongkorn University^32^. The software architecture was three-tiered i.e. client, application server, and database server. The client section was initially constructed for Android OS using Java language. The application server was developed by using JavaScript which responded to user requests and monitored the types of data that should be recorded in the database server. All processes were tested accurately in both software testing and acceptance testing by developers and research team to ensure that the application can function in real settings.

In this study, the application was initially designed to target high-school students who have a smartphone and presumably have good knowledge of rabies. The application was developed in collaboration with the Department of Livestock Development (DLD), Ministry of Agriculture and Cooperatives Thailand, who are responsible for rabies control in Thailand. The application comprised three main sections: (i) demographic information about a dog’s owner, (ii) demographic information about dogs, and (iii) routine information updates and report management. The first and second sections were recorded in literal format once for each dog and owner upon registration. Monthly updates were required to follow-up on status of registered dogs, e.g. still alive, moved out, dead, vaccination status, and sterilization status. The participants were reminded to provide at least the monthly updates through the application and other channels of communication including Line messaging application and telephone calls with the researchers.

#### Qualitative study for the evaluation of participatory research

The second part of the study was conducted once the dog survey had been completed. This qualitative study aimed to explore in detail the knowledge, perceptions, and awareness of dog owners with regard to rabies in dogs and humans. Semi-structured interviews were used to collect the information. First, the participants from the survey were asked to voluntarily participate in the qualitative study by registering online to express their interest. To ensure a diversity of data, the research team purposively selected participants to include students whose duration of participation in the dog survey varied and those who attended different schools. Second, they were invited to participate in a one-to-one online interview with Thichumpa W. Each interview lasted for 15–30 min and was recorded. Informed consent was obtained from all participants’ parents. The interviews were conducted between March to May 2021.

The study protocol was approved by the ethical committees of Mahidol University Central Institutional Review Board (MU-CIRB 2019/157.0606; August 2019). Written informed consent was obtained from all high school students who participated in the research. All the methods were performed in accordance with relevant guidelines and regulations.

#### Data analyzes

Descriptive statistics were generated using SPSS version 23.0^33^. For the qualitative study, transcript data were evaluated by determining the frequency of answers given by interviewees and then coding keywords into pre-set themes^34^, including the theme of rabies knowledge, rabies awareness, caring for owned dog, perception about project, and other suggestions. The content analysis and thematic narrative approach were performed using QDA Miner Lite^35^.

## Supplementary Information

Supplementary Tables.

## Data Availability

The data that support the findings of this study are available from the corresponding author, (WP), upon reasonable request.
