# Supplementary material for: Study of dog population dynamics and rabies awareness in Thailand using a school-based participatory research approach
Source: Sci Rep. 2024 Sep 3;14:20477. doi: 10.1038/s41598-024-71207-7 (PMC11372070; doi:10.1038/s41598-024-71207-7)
Supplement: Supplementary file 1 — Supplementary Tables. [file 41598_2024_71207_MOESM1_ESM.docx]

**Supplementary Material**

**Supplementary Table1**: Dog demographic divided by sexes and study sites

| **Dog data (n=322)** | **Number (%)** | | **Total** | **Male:Female ratio** |
| --- | --- | --- | --- | --- |
| **Area** | **Female** | **Male** |  |  |
| Surin | 41(12.7) | 73(22.7) | 114 | 1.7:1 |
| Chiang Rai | 31(9.6) | 61(18.9) | 92 | 1.9:1 |
| Chon Buri | 31(9.6) | 46(14.2) | 73 | 1.5:1 |
| Songkhla | 10(3.1) | 29(9.0) | 39 | 2.9:1 |
| **Total** | **113** | **209** | **322** | **1.8:1** |

**Supplementary Table2**: Dog population dynamics divided by study sites*

| **Dynamics (n=322)** | **Number** | **%** |
| --- | --- | --- |
| **Newborn** | **24** | **7.5** |
| Surin | 16 | 5.0 |
| Chiang Rai | 0 | 0.0 |
| Chon Buri | 8 | 2.5 |
| Songkhla | 0 | 0.0 |
|  |  |  |
| **Died** | **20** | **6.2** |
| Surin | 16 | 5.0 |
| Chiang Rai | 1 | 0.3 |
| Chon Buri | 3 | 0.9 |
| Songkhla | 0 | 0.0 |
|  |  |  |
| **Missing** | **9** | **2.7** |
| Surin | 2 | 0.6 |
| Chiang Rai | 2 | 0.6 |
| Chon Buri | 5 | 1.6 |
| Songkhla | 0 | 0.0 |

*Data on sexes are not available

**Supplementary Table3**: Interview outcomes (N = 23, including 4 students with registration data only, 9 students with registration and some monthly updates and 10 with registration and 12-monthly updates)

|  | **Theme** | **Response code** | **Frequency of answer**  **(n=23)** |  | **Participation level** | | |
| --- | --- | --- | --- | --- | --- | --- | --- |
|  |  |  |  | **%** | **no**  **(n=4)** | **partial**  **(n=9)** | **full**  **(n=10)** |
| 1. | Rabies knowledge |  |  |  |  |  |  |
|  | a. rabies situation in study area | do not know/not follow information | 12 | 52.2 | 1 | 4 | 6 |
|  |  | normal/no cases/rare case | 11 | 47.8 | 3 | 5 | 4 |
|  |  |  |  |  |  |  |  |
|  | b. rabies severity | not sure | 2 | 8.7 |  | 1 | 1 |
|  |  | cause of death | 21 | 91.3 | 4 | 8 | 9 |
|  |  |  |  |  |  |  |  |
|  | c. reservoirs (multiple answers) | only cat and dog | 15 | 65.2 | 3 | 8 | 4 |
|  |  | only dog and stray dog | 4 | 17.4 | 1 |  | 3 |
|  |  | only stray cat and stray dog | 1 | 4.3 |  |  | 1 |
|  |  | mammals | 2 | 8.7 |  | 1 | 1 |
|  |  | rat | 2 | 8.7 |  |  | 2 |
|  |  | cattle | 1 | 4.3 | 1 |  |  |
|  |  | monkey | 1 | 4.3 | 1 |  |  |
|  |  | rabbit | 1 | 4.3 |  |  | 1 |
|  |  | unvaccinated animals | 1 | 4.3 |  |  | 1 |
|  |  | not sure for other animals | 18 | 78.3 | 3 | 8 | 7 |
|  | d. rabies information source | social media | 16 | 69.6 | 3 | 5 | 8 |
|  |  | internet | 15 | 65.2 | 2 | 5 | 8 |
|  |  | school (project visit) | 10 | 43.5 | 1 | 5 | 4 |
|  |  | Television and news | 3 | 13.0 |  | 1 | 2 |
|  |  | community announcement | 1 | 4.3 | 1 |  |  |
|  |  | medical providers | 1 | 4.3 |  | 1 |  |
|  |  | parent | 1 | 4.3 |  |  | 1 |
|  |  | relatives | 1 | 4.3 |  |  | 1 |
|  |  |  |  |  |  |  |  |
| 2. | Rabies awareness |  |  |  |  |  |  |
|  | a. prevention methods of family members | avoid stray dogs | 20 | 87.0 | 2 | 9 | 9 |
|  |  | annual dog vaccination | 12 | 52.2 | 1 | 5 | 6 |
|  |  | observe dog abnormality | 4 | 17.4 | 1 | 1 | 2 |
|  |  | beware of dog bite | 4 | 17.4 | 1 | 2 | 1 |
|  |  | avoid dog saliva | 3 | 13.0 |  | 3 |  |
|  |  | dog confinement | 2 | 8.7 | 1 | 1 |  |
|  |  | hand hygiene after petting | 1 | 4.3 |  | 1 | 1 |
|  |  | beware scratching | 1 | 4.3 | 1 |  |  |
|  |  | vaccination after getting bite/scratch | 1 | 4.3 |  | 1 |  |
|  |  | do not agitate stray dogs | 1 | 4.3 |  |  | 1 |
|  | b. control of dog contact | confinement | 20 | 87.0 | 4 | 7 | 9 |
|  |  | dog leash | 2 | 8.7 |  | 2 |  |
|  |  | dog muzzle | 1 | 4.3 |  |  | 2 |
|  |  | dog cage | 1 | 4.3 | 1 |  |  |
|  |  | using dog warning sign | 1 | 4.3 |  |  | 1 |
|  |  | do nothing | 1 | 4.3 |  |  | 1 |
|  | c. dog interventions for rabies prevention | partially help/unvaccinated dog outside | 11 | 47.8 | 1 | 5 | 5 |
|  |  | can help a lot for reduce spreading | 7 | 30.4 | 1 | 2 | 4 |
|  |  | can help/reducing contact after sterilization | 1 | 4.3 |  | 1 |  |
|  |  | not sure | 1 | 4.3 |  | 1 |  |
|  | e. Confidence level of owners that owned dogs were rabies-free | 30 percent | 1 | 4.3 |  |  | 1 |
|  |  | 50 percent | 1 | 4.3 |  |  | 1 |
|  |  | 60 percent | 2 | 8.7 |  | 2 |  |
|  |  | 70 percent | 7 | 30.4 | 3 | 1 | 3 |
|  |  | 80 percent | 5 | 21.7 | 1 | 2 | 2 |
|  |  | 90 percent | 4 | 17.4 |  | 3 | 1 |
|  |  | 100 percent | 3 | 13.0 |  | 1 | 2 |
|  |  |  |  |  |  |  |  |
| 3. | Caring for owned dog |  |  |  |  |  |  |
|  | a. dog health and illness | animal clinic | 20 | 87.0 | 3 | 7 | 10 |
|  |  | animal hospital | 5 | 21.7 | 1 | 1 | 3 |
|  |  | treated by owner (one give paracetamol) | 5 | 21.7 |  | 3 | 2 |
|  |  | government veterinary service | 3 | 13.0 | 2 |  | 1 |
|  | b. main owner | parents | 16 | 69.6 | 4 | 6 | 6 |
|  |  | grandfather/grandmother | 4 | 17.4 |  | 1 | 3 |
|  |  | aunt | 3 | 13.0 |  | 1 | 2 |
|  |  | student | 1 | 4.3 |  | 1 |  |
|  |  |  |  |  |  |  |  |
|  | c. management of newborns | distribution | 15 | 65.2 | 4 | 5 | 6 |
|  |  | owned by owner | 9 | 39.1 | 1 | 3 | 5 |
|  |  | sell | 1 | 4.3 |  |  | 1 |
|  |  | have no idea | 4 | 17.4 |  | 2 | 2 |
|  | d. management of a dog in heat | dog confinement | 16 | 69.6 | 2 | 7 | 7 |
|  |  | using dog cage | 1 | 4.3 | 1 |  |  |
|  |  | chase other dogs | 2 | 8.7 | 1 | 1 |  |
|  |  | let dog get away from other dogs | 2 | 8.7 | 1 | 1 |  |
|  |  | chained dog | 4 | 17.4 | 1 | 2 | 1 |
|  |  | do nothing/allow to breed | 2 | 8.7 |  | 1 | 1 |
|  |  | neutering before allowing to breed | 2 | 8.7 | 1 | 1 |  |
|  | f. dog observation | eating time | 11 | 47.8 | 1 | 5 | 5 |
|  |  | sleeping time | 7 | 30.4 | 1 | 3 | 3 |
|  |  | playing observation | 4 | 17.4 | 1 | 1 | 2 |
|  |  | any abnormalities | 4 | 17.4 |  | 2 | 2 |
|  |  |  |  |  |  |  |  |
| 4. | Perception about project |  |  |  |  |  |  |
|  | a. barriers to data update | forgot to update | 15 | 65.2 | 3 | 6 | 6 |
|  |  | school assignments and portfolios | 7 | 30.4 | 2 | 1 | 4 |
|  |  | part time jobs | 4 | 17.4 | 1 | 1 | 2 |
|  |  | personal works | 4 | 17.4 | 1 |  | 3 |
|  |  | internet connection | 3 | 13.0 | 2 |  | 1 |
|  |  | changed smart phone | 2 | 8.7 | 1 | 1 |  |
|  |  | cannot install Pupify | 1 | 4.3 | 1 |  |  |
|  |  | not follow and interest | 1 | 4.3 | 1 |  |  |
|  | b. motivation | certificate | 14 | 60.9 |  | 7 | 7 |
|  |  | project reward for data update | 8 | 34.8 |  | 2 | 6 |
|  |  | research experience | 3 | 13.0 |  | 3 |  |
|  |  | dog care and follow up | 3 | 13.0 |  | 3 |  |
|  |  | rabies information | 1 | 4.3 |  |  | 1 |
|  | c. project advantage | dog attention and care | 16 | 69.6 |  | 8 | 8 |
|  |  | dog behavior observation | 8 | 34.8 |  | 4 | 4 |
|  |  | dog vaccine notification | 4 | 17.4 |  | 1 | 3 |
|  |  | education | 4 | 17.4 |  | 2 | 2 |
|  | d. key influencer | own self | 15 | 65.2 | 1 | 6 | 8 |
|  |  | project notification | 3 | 13.0 |  | 1 | 2 |
|  |  | project reward | 2 | 8.7 |  | 2 |  |
|  |  | school activity support | 1 | 4.3 |  | 1 |  |
|  |  |  |  |  |  |  |  |
| 5. | Suggestion | necessary information in Pupify | 2 | 8.7 | 1 |  | 1 |
|  |  | social media/fanpage for contacting new generation/participants | 2 | 8.7 |  |  | 2 |
|  |  | education of dog management | 1 | 4.3 |  |  | 1 |
|  |  | education of vaccination | 1 | 4.3 |  |  | 1 |
|  |  | add more information/dog treat and prevention | 1 | 4.3 |  | 1 |  |
|  |  | owner responsibility is important | 1 | 4.3 |  |  | 1 |
|  |  | add infographic | 1 | 4.3 |  |  | 1 |
